# Supplementary material for: The impact of the UK soft drink industry levy on the soft drink marketplace, 2017–2020: An interrupted time series analysis with comparator series
Source: PLoS One. 2024 Jun 6;19(6):e0301890. doi: 10.1371/journal.pone.0301890 (PMC11156274; doi:10.1371/journal.pone.0301890)
Supplement: S1 File — (PDF) [file pone.0301890.s001.pdf]

S1 File. Full regression outputs and sensitivity analysis

S1 Table. Full regression outputs for models of reformulation (sugar per 100mL used in drinks), volume, price, and use of non-sugar sweeteners.

Model	Parameter	Coefficient	Standard Error	p-value	Lower 95% CI	Upper 95% CI
Proportion of comparator drinks that have below 5g sugar/100mL	Intercept	0.246	0.002	<0.0001	0.242	0.249
	Time	0.000	0.000	0.004	0.000	0.000
	Intervention	0.009	0.011	0.432	-0.013	0.031
	1st Oct 18 – 31st Mar 19 (Period 3)	0.005	0.015	0.716	-0.024	0.034
	1st Apr 19 – 30th Sep 19 (Period 4)	0.012	0.028	0.674	-0.043	0.067
	1st Oct 19 – Mar 20 (Period 5)	0.003	0.033	0.928	-0.061	0.067
	Christmas (December)	-0.003	0.003	0.326	-0.008	0.003
	Summer Months (June, July, and August)	-0.004	0.011	0.730	-0.025	0.017
	Time*Intervention	0.000	0.000	0.748	0.000	0.000
Proportion of eligible drinks that have below 5g sugar/100mL	Intercept	0.773	0.015	<0.0001	0.744	0.801
	Time	0.001	0.000	0.0005	0.000	0.001
	Intervention	0.078	0.020	0.0001	0.038	0.117
	1st Oct 18 – 31st Mar 19 (Period 3)	0.009	0.006	0.148	-0.003	0.021
	1st Apr 19 – 30th Sep 19 (Period 4)	-0.006	0.015	0.667	-0.036	0.023
	1st Oct 19 – Mar 20 (Period 5)	-0.014	0.017	0.416	-0.047	0.019
	Christmas (December)	0.003	0.003	0.291	-0.003	0.010
	Summer Months (June, July, and August)	-0.001	0.005	0.850	-0.011	0.009
	Time*Intervention	0.000	0.000	0.003	-0.001	0.000
Proportion of comparator drinks that have 8g sugar/100mL or more	Intercept	0.665	0.001	<0.0001	0.663	0.668
	Time	0.000	0.000	<0.0001	0.000	0.000
	Intervention	-0.014	0.008	0.095	-0.030	0.002
	1st Oct 18 – 31st Mar 19 (Period 3)	-0.015	0.011	0.167	-0.036	0.006
	1st Apr 19 – 30th Sep 19 (Period 4)	-0.019	0.018	0.295	-0.055	0.017

	1st Oct 19 – Mar 20 (Period 5)	-0.010	0.024	0.660	-0.057	0.036
	Christmas (December)	0.002	0.002	0.297	-0.001	0.005
	Summer Months (June, July, and August)	0.003	0.006	0.597	-0.008	0.014
	Time*Intervention	0.000	0.000	0.006	0.000	0.000
Proportion of eligible drinks that have 8g sugar/100mL or more	Intercept	0.133	0.012	<0.0001	0.108	0.157
	Time	0.000	0.000	0.004	-0.001	0.000
	Intervention	-0.063	0.019	0.001	-0.100	-0.026
	1st Oct 18 – 31st Mar 19 (Period 3)	-0.010	0.006	0.075	-0.022	0.001
	1st Apr 19 – 30th Sep 19 (Period 4)	0.000	0.011	0.981	-0.023	0.022
	1st Oct 19 – Mar 20 (Period 5)	0.008	0.015	0.572	-0.021	0.037
	Christmas (December)	-0.005	0.002	0.046	-0.010	0.000
	Summer Months (June, July, and August)	0.000	0.005	0.924	-0.010	0.009
	Time*Intervention	0.000	0.000	0.007	0.000	0.001
Volume of eligible drinks that have less than 5g sugar/100mL	Intercept	1297.72	4.85	<0.0001	1288.21	1307.23
	Time	0.14	0.05	0.010	0.03	0.24
	Intervention	-12.15	12.52	0.332	-36.68	12.39
	1st Oct 18 – 31st Mar 19 (Period 3)	-30.28	9.47	0.001	-48.84	-11.73
	1st Apr 19 – 30th Sep 19 (Period 4)	-28.65	18.76	0.127	-65.42	8.13
	1st Oct 19 – Mar 20 (Period 5)	-4.89	25.65	0.849	-55.17	45.39
	Christmas (December)	0.06	4.15	0.988	-8.06	8.19
	Summer Months (June, July, and August)	-16.81	8.12	0.038	-32.72	-0.90
	Time*Intervention	-0.03	0.07	0.698	-0.17	0.11
Volume of eligible drinks that have 8g sugar/100mL or more	Intercept	1202.74	105.93	<0.0001	995.11	1410.36
	Time	-1.69	1.10	0.125	-3.85	0.47
	Intervention	-305.12	104.92	0.004	-510.75	-99.49
	1st Oct 18 – 31st Mar 19 (Period 3)	-54.16	34.52	0.117	-121.83	13.50

	1st Apr 19 – 30th Sep 19 (Period 4)	-78.74	46.20	0.088	-169.29	11.81
	1st Oct 19 – Mar 20 (Period 5)	-18.10	90.27	0.841	-195.02	158.82
	Christmas (December)	-21.09	25.27	0.404	-70.61	28.44
	Summer Months (June, July, and August)	-13.47	17.65	0.445	-48.07	21.12
	Time*Intervention	1.93	1.12	0.084	-0.26	4.12
Volume of eligible drinks that have 5 up to 8g of sugar/100mL	Intercept	796.69	3.61	<0.0001	789.62	803.77
	Time	0.00	0.08	0.951	-0.15	0.14
	Intervention	-7.56	16.13	0.639	-39.18	24.05
	1st Oct 18 – 31st Mar 19 (Period 3)	21.79	52.95	0.681	-81.99	125.58
	1st Apr 19 – 30th Sep 19 (Period 4)	42.46	71.48	0.553	-97.64	182.56
	1st Oct 19 – Mar 20 (Period 5)	-57.19	117.60	0.627	-287.69	173.32
	Christmas (December)	-5.77	9.46	0.542	-24.31	12.77
	Summer Months (June, July, and August)	-42.41	21.11	0.045	-83.79	-1.04
	Time*Intervention	0.02	0.15	0.896	-0.28	0.32
Volume of exempt drinks	Intercept	1016.90	2.35	<0.0001	1012.30	1021.51
	Time	-0.04	0.03	0.182	-0.10	0.02
	Intervention	-5.04	5.21	0.332	-15.25	5.16
	1st Oct 18 – 31st Mar 19 (Period 3)	-46.47	9.55	<0.0001	-65.19	-27.75
	1st Apr 19 – 30th Sep 19 (Period 4)	-39.19	12.31	0.001	-63.32	-15.06
	1st Oct 19 – Mar 20 (Period 5)	-28.94	17.89	0.106	-64.01	6.13
	Christmas (December)	0.78	2.52	0.758	-4.16	5.71
	Summer Months (June, July, and August)	-8.31	5.28	0.115	-18.66	2.04
	Time*Intervention	0.07	0.04	0.095	-0.01	0.15
Price of eligible drinks in no tax levy (have 5g sugar/100mL or less)	Intercept	0.2121	0.0022	<0.0001	0.2078	0.2163
	Time	-0.0001	0.0000	0.021	-0.0001	0.0000
	Intervention	0.0120	0.0057	0.035	0.0008	0.0232

	1st Oct 18 – 31st Mar 19 (Period 3)	-0.0175	0.0084	0.037	-0.0338	-0.0011
	1st Apr 19 – 30th Sep 19 (Period 4)	-0.0189	0.0114	0.097	-0.0412	0.0034
	1st Oct 19 – Mar 20 (Period 5)	-0.0137	0.0156	0.381	-0.0443	0.0169
	Christmas (December)	-0.0081	0.0019	<0.0001	-0.0118	-0.0045
	Summer Months (June, July, and August)	0.0083	0.0047	0.079	-0.0009	0.0175
	Time*Intervention	0.0001	0.0000	0.121	0.0000	0.0001
Price of eligible drinks in higher tax levy group (8g sugar/100 mL or more)	Intercept	0.2509	0.0083	<0.0001	0.2345	0.2672
	Time	0.0001	0.0001	0.456	-0.0001	0.0003
	Intervention	0.0494	0.0079	<0.0001	0.0339	0.0649
	1st Oct 18 – 31st Mar 19 (Period 3)	-0.0027	0.0070	0.702	-0.0164	0.0110
	1st Apr 19 – 30th Sep 19 (Period 4)	-0.0017	0.0096	0.855	-0.0205	0.0170
	1st Oct 19 – Mar 20 (Period 5)	0.0102	0.0132	0.443	-0.0158	0.0361
	Christmas (December)	-0.0008	0.0060	0.888	-0.0126	0.0109
	Summer Months (June, July, and August)	0.0047	0.0034	0.172	-0.0020	0.0113
	Time*Intervention	0.0000	0.0001	0.827	-0.0002	0.0002
Price of eligible drinks in lower tax levy group (5g up to 8g of sugar/100mL)	Intercept	0.2937	0.0063	<0.0001	0.2814	0.3060
	Time	-0.0004	0.0001	0.002	-0.0007	-0.0002
	Intervention	0.0091	0.0193	0.639	-0.0288	0.0470
	1st Oct 18 – 31st Mar 19 (Period 3)	0.0001	0.0267	0.998	-0.0523	0.0525
	1st Apr 19 – 30th Sep 19 (Period 4)	-0.0210	0.0403	0.601	-0.1000	0.0579
	1st Oct 19 – Mar 20 (Period 5)	0.0356	0.0555	0.521	-0.0731	0.1443
	Christmas (December)	-0.0480	0.0144	0.001	-0.0763	-0.0197
	Summer Months (June, July, and August)	0.0329	0.0140	0.019	0.0055	0.0603
	Time*Intervention	0.0005	0.0002	0.001	0.0002	0.0008
Price of exempt drinks	Intercept	0.2348	0.0032	<0.0001	0.2286	0.2410
	Time	0.0000	0.0000	0.497	0.0000	0.0001

	Intervention	-0.0041	0.0050	0.411	-0.0140	0.0057
	1st Oct 18 – 31st Mar 19 (Period 3)	0.0227	0.0083	0.006	0.0064	0.0389
	1st Apr 19 – 30th Sep 19 (Period 4)	0.0231	0.0138	0.095	-0.0040	0.0501
	1st Oct 19 – Mar 20 (Period 5)	0.0291	0.0203	0.152	-0.0107	0.0689
	Christmas (December)	0.0032	0.0021	0.134	-0.0010	0.0073
	Summer Months (June, July, and August)	0.0051	0.0034	0.132	-0.0015	0.0118
	Time*Intervention	-0.0001	0.0000	0.130	-0.0002	0.0000
Non-nutritive sweetener use in eligible drinks	Intercept	0.744	0.006	<0.0001	0.732	0.755
	Time	0.000	0.000	0.025	0.000	0.000
	Intervention	0.043	0.011	<0.0001	0.022	0.063
	1st Oct 18 – 31st Mar 19 (Period 3)	-0.013	0.012	0.262	-0.036	0.010
	1st Apr 19 – 30th Sep 19 (Period 4)	-0.020	0.018	0.278	-0.055	0.016
	1st Oct 19 – Mar 20 (Period 5)	-0.035	0.023	0.129	-0.080	0.010
	Christmas (December)	-0.009	0.006	0.166	-0.021	0.004
	Summer Months (June, July, and August)	-0.002	0.006	0.734	-0.015	0.010
	Time*Intervention	0.000	0.000	0.204	0.000	0.000
Non-Nutritive Sweeteners in comparator group	Intercept	0.017	0.001	<0.0001	0.015	0.018
	Time	0.00004	0.000	<0.0001	0.000	0.000
	Intervention	0.000	0.002	0.839	-0.004	0.003
	1st Oct 18 – 31st Mar 19 (Period 3)	-0.001	0.002	0.578	-0.006	0.003
	1st Apr 19 – 30th Sep 19 (Period 4)	-0.010	0.005	0.031	-0.020	-0.001
	1st Oct 19 – Mar 20 (Period 5)	-0.010	0.007	0.151	-0.024	0.004
	Christmas (December)	0.000	0.001	0.741	-0.001	0.002
	Summer Months (June, July, and August)	-0.003	0.001	0.054	-0.005	0.000
	Time*Intervention	-0.00004	0.000	0.003	0.000	0.000

S2 Table. Sensitivity analysis for price, using the lowest possible price per 100mL based on promotional deals.

Model	Parameter	Coefficient	Standard Error	p-value	Lower 95% CI	Upper 95% CI
Price of Products (with full promotion possible applied) in no tax levy group	Intercept	0.2044	0.0021	<0.0001	0.2002	0.2086
	Time	-0.0001	0.0000	0.080	-0.0001	0.0000
	Intervention	0.0114	0.0065	0.078	-0.0013	0.0242
	1st Oct 18 – 31st Mar 19 (Period 3)	-0.0167	0.0105	0.113	-0.0374	0.0040
	1st Apr 19 – 30th Sep 19 (Period 4)	-0.0204	0.0150	0.172	-0.0498	0.0089
	1st Oct 19 – Mar 20 (Period 5)	-0.0168	0.0188	0.373	-0.0537	0.0201
	Christmas (December)	-0.0090	0.0020	<0.0001	-0.0129	-0.0052
	Summer Months (June, July, and August)	0.0095	0.0054	0.079	-0.0011	0.0202
	Time*Intervention	0.0000	0.0000	0.276	0.0000	0.0001
Price of Products (with full promotion possible applied) in higher tax levy group	Intercept	0.2419	0.0062	<0.0001	0.2297	0.2540
	Time	0.0001	0.0001	0.141	0.0000	0.0003
	Intervention	0.0314	0.0062	<0.0001	0.0192	0.0435
	1st Oct 18 – 31st Mar 19 (Period 3)	-0.0096	0.0113	0.396	-0.0316	0.0125
	1st Apr 19 – 30th Sep 19 (Period 4)	-0.0150	0.0139	0.279	-0.0422	0.0122
	1st Oct 19 – Mar 20 (Period 5)	-0.0057	0.0201	0.775	-0.0451	0.0336
	Christmas (December)	-0.0011	0.0098	0.908	-0.0204	0.0181
	Summer Months (June, July, and August)	0.0089	0.0037	0.016	0.0016	0.0162
	Time*Intervention	-0.0001	0.0001	0.399	-0.0003	0.0001
Price of Products (with full promotion possible applied) in lower tax levy group	Intercept	0.2745	0.0065	<0.0001	0.2619	0.2872
	Time	-0.0004	0.0001	0.001	-0.0007	-0.0002
	Intervention	0.0101	0.0146	0.488	-0.0185	0.0388
	1st Oct 18 – 31st Mar 19 (Period 3)	0.0040	0.0232	0.864	-0.0415	0.0494
	1st Apr 19 – 30th Sep 19 (Period 4)	-0.0238	0.0326	0.466	-0.0878	0.0402
	1st Oct 19 – Mar 20 (Period 5)	0.0269	0.0459	0.557	-0.0631	0.1170
	Christmas (December)	-0.0503	0.0136	0.0002	-0.0770	-0.0236
	Summer Months (June, July, and August)	0.0366	0.0128	0.004	0.0115	0.0617
	Time*Intervention	0.0005	0.0001	0.0002	0.0002	0.0008
Price of Products (with full promotion	Intercept	0.2003	0.0020	<0.0001	0.1963	0.2042
	Time	-0.0001	0.0000	0.062	-0.0001	0.0000
	Intervention	0.0007	0.0029	0.814	-0.0050	0.0063

possible applied) in comparator group	1st Oct 18 – 31st Mar 19 (Period 3)	0.0153	0.0046	0.001	0.0064	0.0243
	1st Apr 19 – 30th Sep 19 (Period 4)	0.0076	0.0072	0.287	-0.0064	0.0217
	1st Oct 19 – Mar 20 (Period 5)	0.0072	0.0123	0.555	-0.0168	0.0313
	Christmas (December)	-0.0043	0.0024	0.077	-0.0091	0.0005
	Summer Months (June, July, and August)	0.0074	0.0026	0.004	0.0023	0.0124
	Time*Intervention	0.0001	0.0000	0.153	0.0000	0.0001
