# Supplementary material for: The impact of the UK soft drink industry levy on the soft drink marketplace, 2017–2020: An interrupted time series analysis with comparator series
Source: PLoS One. 2024 Jun 6;19(6):e0301890. doi: 10.1371/journal.pone.0301890 (PMC11156274; doi:10.1371/journal.pone.0301890)
Supplement: Attachment — Submitted filename: SDIL_reviewer_comments_FINAL.docx [file pone.0301890.s002.docx]

**The impact of the UK Soft Drink Industry Levy on the soft drink marketplace, 2017 – 2020: an interrupted time series analysis with comparator series**

Response to reviewer comments

Please find our response to the reviewers’ comments below. Our responses are provided in blue.

Reviewer #1: Overview
This paper uses interrupted time series analyses to evaluate the effects of the UK soft drink industry levy on sugar levels, price, volume, and non-sugar sweeteners. This is an important topic, and the authors provide strong justification for their study, which examines medium-term impacts of the intervention. The paper is interesting, well written and thorough. I have some concerns about the specification of the interrupted time series models, and have made some suggestions for the authors to consider and respond to. In particular, I suggest that the 6-monthly breakpoints in all models should be further justified or removed.

We thank the reviewer for their helpful comments on our paper.

Abstract and Introduction: These sections are clear, with good justification provided for the study.

No response needed.

Outcome Measures: The definitions for time series would benefit from additional detail. For all measures, how was SKU-level information combined? My best guess is that it is the proportion of soft drink SKUs, and the average across soft drink SKUs (with each SKU weighted equally regardless of volume).

Thank you for your comment. We have clarified this in the outcomes section by adding the following sentence:

*Items were considered by SKU, so those outcome measures which are a proportion, were the proportion of all SKU on a given date, and those that are averages were the average of all SKU on the given date.*

Statistical Methods: I am not fully convinced by the model specification of splitting the data into six month periods. I think this is essentially testing for a series of arbitrary breakpoints that are unlikely to best fit the series. From the figures, they do not look like they fit the data well. Are there any statistical papers recommending this approach?

In my view the specification should reflect the expected structure of the series – which is more likely to be a step and a slope change at the intervention point only. If the authors expect that the intervention effect will have decayed by the end of the series, and do not want late datapoints to influence the intervention effects, they might consider using a pulse effect instead of a slope change, perhaps as a sensitivity analysis e.g. <https://www.ncbi.nlm.nih.gov/books/NBK563546/>. If breakpoints are identified on visual inspection of the series, I would suggest including them, but only if they are significant and improve model parsimony.

The reviewer raises some very important points in the two previous comments, which are related and so we have addressed them together. Several commentary pieces have described challenges to reducing biases in ITS analyses. These challenges include short pre-intervention time periods which can make it difficult to determine secular trends, and having too many data points in the post-intervention period, which can influence outcome variables if the secular trend has changed with time (Bernal et al. 2017, Pape et al. 2013, Penfold and Zhang 2013, Ramsay et al. 2003). We are not aware of specific guidance or precedents in the peer-reviewed literature for our approach of breaking the post-intervention period into four equally sized periods. However, we believe that we have good justification to do so, which we present below.

Our first principle was that we wanted to set the models to test hypotheses, rather than to find the closest fit to the observed data points. Our intention here was to use the specific case of the UK SDIL to derive generalisable findings about how the food industry reacts to economic stimuli, rather than to produce results that are highly specific to the effects observed in the UK retail marketplace for this specific intervention. In a previous paper of ours (Scarborough et al., 2020) we took the opposite approach and developed data-driven models with the aim of matching trends as closely as possible - our learning from that experience was that there are so many potential specifications of an ITS model that it is very easy to find spurious patterns in the data if you take a purely data-driven approach.

Our second principle was that we wanted to have a single model structure that we would apply across all of the analyses that we conducted in this paper (due to multiple outcomes and multiple subgroups of drinks, there are 14 analyses conducted in this paper before sensitivity analyses are taken into account). We had two reasons for this approach: first, so that results were directly comparable between different analyses, and second to ease reporting and understanding of the model results.

Our third principle was that we wanted to protect against undue influence of data points that occur far from the intervention. This was a particular problem for our analyses, since we had six months of data before the intervention and nearly two years after the intervention. The reviewer suggests that we could have used a pulse analysis (which assumes that the effect of the intervention is limited to only a small amount of time after the intervention takes place) to protect against this undue influence. We considered this, but we did not think that a pulse analysis was an accurate representation of our hypothesis, which was that the SDIL has had a lasting effect on manufacturer and retailer behaviour over the two years after implementation of the SDIL. However, even though our hypothesis suggested that the data from two years after the implementation should influence the results, there are still methodological reasons for wanting to correct for *undue* influence of the effect of later timepoints. If we had conducted a model with a slope and step change at the point of the intervention, but without any break points after the intervention, then small deviations in outcomes far from the intervention would have had far greater influence on the resultant model than those that are closer to the intervention (since small changes in the modelled slope have little effect on residuals for points close to the intervention, but a big difference in residuals for points far from the intervention where the effect of the slope change has compounded over time). Therefore, we decided to add arbitrary break points after the intervention to protect against this influence. We chose to break our post-intervention period into four periods of approximately equal size. For the reasons described above, we used this same model structure across all models (rather than selecting break points based on observations of the data).

We recognise that our model selection was not the only option. As the reviewer suggests, we could have built models with a slope and step change at the point of intervention and no break points after the intervention. We also could have allowed a data-driven approach to select break points after the intervention using a statistical package that tests all timepoints post-intervention and allows additional breakpoints to be added only if predefined criteria are met. To demonstrate how our model selection affected outcomes we ran a sensitivity analysis which compared our original model specification with the two options described above. We selected the model that looked at volume change in high sugar level drinks for our sensitivity analysis as that model showed the most dramatic change in trend post-intervention. The results of this sensitivity analysis are shown in the figure below.

**Figure:** Sensitivity analysis comparing three different model specifications for the ‘volume change in high sugar drinks’ model. Panel (A) shows the full results, and panel (B) magnifies the post-intervention results to highlight differences.

As can be observed from the figure, the results of the three different model specifications all produce the same conclusion – that there was a large step change in volume of high sugar drinks at the implementation of the SDIL, and since then volumes of high sugar drinks have begun to return to pre-SDIL levels. In this instance, our pre-specified model selection predicted a more conservative reduction in volume immediately after the intervention than the model with no breakpoints. The model with the data-driven post-intervention breakpoint suggests that the increase in volume of high sugar drinks occurred later (and was more dramatic) than the other two models.

We do not claim that our model specification is more appropriate than the other two that have been implemented in the sensitivity analysis. However, we believe the sensitivity analysis does demonstrate that the choice of model specification is not likely to have had a major impact on the conclusions of the paper. Because of this (and because of the justifications provided earlier in this response), we see no reason to move away from our pre-specified approach for the paper.

As can be seen from this discussion, the justification for our model selection is nuanced and is difficult to fully capture in a short summary. However, we have tried to provide some of this justification in the methods section of the paper, by adding the following text:

*We decided to use the same model structure for all models and outcomes tested in this paper for ease of reporting and to facilitate comparisons between results. Our model specification prioritised hypothesis testing over reproduction of observed trends in the data to increase the generalisability of the research findings.*

Additional methods queries:
1. I wondered how changes to the Tesco clubcard scheme might have affected prices e.g. the launch of clubcard plus and clubcard prices in 2019. The authors may want to consider controlling for these changes.

The clubcard prices were announced in 2019, which includes some of the time period of our study, but it was only [trialled for a month](https://www.tescoplc.com/tesco-introduces-special-clubcard-prices-for-customers-to-celebrate-100-years-of-great-value/) initially and then was [launched more widely in September 2020](https://www.moneysavingexpert.com/news/2020/09/tesco--clubcard-prices--are-back-again---but-are-they-any-good-/), which is outside of the time frame of our study.

In one of our sensitivity analyses reported in the paper, we consider how the analysis might change if we assumed that price promotions were taken into account. This would take into account the possible promotions available with a clubcard during this one month trial, as well as other available promotions which may have impacted prices.

2. The authors discuss the effect of inflation on the real-term value of the levy over time, and conclude that its effects have not diminished. I think this might be incorrect, as inflation means that similar price levels being maintained means soft drinks have become cheaper over time in real-terms. Perhaps the price analyses should use inflation-adjusted prices?

Much of the inflation in the UK has been seen since late 2020 or early 2021, which is beyond the time of this study (ONS, 2023). For example, the average price of eligible products in the high tax category was £0.35/L on 14 March 2020. Using the Bank of England’s inflation calculator, accounting for inflation, this product should have cost £0.33/L in 2017, but in December 2023 it would cost £0.43/L. During the time period of our study then, the prices were not grossly different, however, the dramatic increase in inflation does raise concerns about the impact of the tax going forward since it has not been adjusted for inflation. This point is already mentioned in the discussion:

*However, since our dataset only extends to March 2020, it does not include the recent sharp increase in food prices observed as a result of the cost-of-living crisis in the UK. Unlike UK tobacco taxes, the SDIL was not index-linked, meaning that the levy does not change as retail prices change.* *As a result, the effective real-terms value of the levy has not kept up with inflation, and each year it will become lower as a proportion of price, and therefore may become less effective. While this did not have a material impact on the findings of this study since inflation remained low during the study period, further analyses are needed on more recent data to measure the impact of the SDIL over the longer term to observe whether this lack of index-linking results in diminishing benefits of the SDIL.*

3. I wondered how lag 7 was chosen for auto-correlation adjustment, and whether any tests were used to confirm that this was sufficient.

As discussed earlier, one of our priorities for model specification was to ensure that all models were comparable. Lag 7 was chosen as it was the lag that would be best fitting for most models after assessing the autocorrelation plots. The lag of some of the models varied, however, as is demonstrated with the Autocorrelation Function (ACF) plots below for ineligible drinks for each of the model outcomes.:

Table 1: I am not highly familiar with the Wilcoxon rank sum test, but some of the significant p-values in Table 1 are confusing to me. For example, in the first row, exempt goes from 8.4 to also 8.4 which has an associated p-value of 0.031. Could the authors explain this?

Thank you for your comment. We used the Wilcoxon rank sum test because prior to the intervention, the distribution of values was not normal (see diagrams below). The Wilcoxon Rank sum test is non-parametric. The null hypothesis for the Wilcoxon rank sum test is that the distribution of the two samples is identical. As can be observed by the distributions below, this is clearly not the case for our data.

Results: It would be very helpful to include a table that summarises all model results for step and slope changes (B, 95% CI, p-value). This could possibly focus on just the effects at the time of the intervention, or could include all effects if the authors apply my suggestions regarding model specifications.

In the supplementary materials we have a table (Supplementary Table 1) that summarises all model results. This table is referenced at the appropriate locations in the text to direct the reader.

Reviewer #2: Thank you for giving me a chance to the submitted manuscript.

Bandy and colleagues examined the midterm impact of the soft drink industry levy (SDIL) on sugar levels, price, portion size and use of non-sugar sweeteners in UK; the data source was obtained from leading retail supermarket and the primary analyses were done by interrupted time series (ITT). The authors found that reductions in sugar levels were sustained by 2020 and a reduction in product volume for higher tier drinks may be diminishing over time.

The manuscript is overall well written, but it has a number of points to be clarified as well.

We thank you for your review and have addressed your comments below.

Major points
The analysis specifically excluded considering slope changes between the various post-implementation time periods (in Statistical Methods section). However, it is noted that these slope changes were subsequently reported, leading to potential confusion. Please explain.

Thank you for your comment. In our model there is an overall slope change that happens at the point of the implementation of the SDIL. However, our model does not allow for any more slope changes at the subsequent breakpoints (only step changes are permitted). We agree that this is confusing and we have amended the methods section to clarify.

Minor points
1. In my understanding, the unit of analysis in this study was the number of available products (not the sales!) at the online supermarket. If this is correct, it would be more appropriate to mention this information earlier in the paper, rather than introducing it for the first time in the limitations section.

Thank you for your comment. We have made sure this is clarified in the methods with the following description:

*This data represents all available offerings on the supermarket website but does not take account of differing sales of drinks.*

2. Abstract: “There was a weak evidence”: the strength of evidence does not depend on p-value alone.

We have revised this part in the abstract, and it now reads:

*The change in trend for the product volume of drinks in the higher levy group between April 2018 and March 2020 was in the increasing direction (+704mL per year, 95%CI: -95, 1504, p = 0.084).*

3. Introduction: Do UK children admit for tooth extraction?

Unfortunately, tooth extraction is the most common reason for elective admissions to hospital for children (aged 5-9) in the UK (Rogers et al, 2023).

4. Methods; The authors have classified diet sodas as eligible drinks. However, it is unclear how the sugar levels in these drinks were determined. As a related issue, conducting sensitivity analyses that reclassify such drinks as non-eligible may be needed.

foodDB provides dietary information for all drinks, so for diet sodas there is information that the soda has low or no sugar. The same method for classification was applied, using the threshold of the tax applied to all drinks which might be eligible (i.e. did not meet the exemption criteria).

We considered classifying diet sodas as non-eligible, but this definition is problematic. We know that the SDIL prompted manufacturers to remove sugar from some of their product range. This means that if we considered low sugar drinks as non-eligible then the same drink (as presented to the consumer) could be considered eligible at the start of the dataset and non-eligible at the end. It also means that a subsample of the ineligible drinks would be affected by the intervention, which limits their usefulness as a comparator series. Finally, we wanted our classifications to be comparable to an earlier published paper on the effect of the SDIL on manufacturer and retailer behaviour (Scarborough et al., 2020).

5. Methods; I am wondering why the authors considered the product volume and price as being for the total product in the case of multipacks.

Thank you for your comment. We considered the product volume and price as the total product because this is how consumers interact with the product. We know (for example) that price per 100mL for the same drink can be different depending on how the drink is sold (in a multipack, in a family size bottle, etc.) Therefore, these different products were considered to be unique items in our dataset (even if they contain the same drink).

6. Methods: I am unclear about the statement, 'it was imputed with the nearest available value for the same product before the missing value.' Could you please clarify if the data were imputed using values from the same product?"

Data were imputed from the same product. We selected a value from the closest point in time where data were available for the same product (e.g. if sugar levels were missing for a 330mL can of Coca-Cola in February 2019, then we would impute sugar levels by taking a value from a 330mL can of Coca-Cola from as close to February 2019 as possible).

7. Figure 1: Hard to visualize.

We appreciate that this is a complicated figure but we feel that it is worthwhile including as much information as possible in Figure 1 to allow it to stand alone. We have also ensured that the colour palette that we are using is acceptable for visually impaired people.

8. Reference: The reference style does not meet the Journal standards.

References have been updated to the Vancouver style.

9. Reference: If available, please cite the URL (eg, TESCO 2023).

The URL has been added to the reference.

10. The manuscript is somewhat lengthy. I think some parts should be move to Supplemental information, including (but not limited to) “Display of results” or “Changes to Protocol”.

There are a lot of results to report in this paper (which is one of the reasons why we sought to keep our model specification comparable across all outcomes – see response above). We have tried to keep the reporting as succinct as possible, but we are happy to move content to the supplementary material if the editors think this is appropriate.

References:

Bernal JL, Cummins S, Gasparrini, A. Interrupted time series regression for the evaluation of public health interventions: a tutorial. International journal of epidemiology, 2017; 46(1), 348–355. https://doi.org/10.1093/ije/dyw098

Office for National Statistics (ONS). Consumer price inflation, UK: November 2023. ONS: Newport, 2023.

Pape UJ, Millett C, Lee JT, Car J, Majeed A. Disentangling secular trends and policy impacts in health studies: use of interrupted time series analysis. Journal of the Royal Society of Medicine, 2013; 106(4), 124–129. https://doi.org/10.1258/jrsm.2012.110319

Penfold RB, Zhang, F. Use of interrupted time series analysis in evaluating health care quality improvements. Academic pediatrics, 2013; 13(6 Suppl), S38–S44. https://doi.org/10.1016/j.acap.2013.08.002

Ramsay CR, Matowe L, Grilli R, Grimshaw JM, Thomas RE. Interrupted time series designs in health technology assessment: lessons from two systematic reviews of behavior change strategies. International journal of technology assessment in health care, 2003; 19(4), 613–623. https://doi.org/10.1017/s0266462303000576

Rogers NT, Conway DI, Mytton OT, Roberts CH, Rutter H, Sherriff A, et al. Trends in childhood hospital admissions for carious tooth extractions in England in relation to the UK soft drink industry levy: an interrupted time series analysis of Hospital Episode Statistics. medRxiv. 2023:2023.02.27.23286504.

Scarborough P, Adhikari V, Harrington RA, Elhussein A, Briggs ADM, Rayner M, Adams J, Cummins S, Penney T, White M. Impact of the announcement and implementation of the UK Soft Drinks Industry Levy on sugar content, price, product size and number of available soft drinks in the UK, 2015-19: a controlled interrupted time series analysis. PLoS Medicine, 2020;17(2):e1003025.
